# Interferon-*γ* suppresses S100A4 transcription independently of apoptosis or cell cycle arrest

**DOI:** 10.1038/sj.bjc.6600998

**Published:** 2003-06-10

**Authors:** K Andersen, B Smith-Sørensen, K B Pedersen, E Hovig, O Myklebost, Ø Fodstad, G M Mælandsmo

**Affiliations:** 1 Department of Tumour Biology, The Norwegian Radium Hospital, Montebello, N-0310 Oslo, Norway

**Keywords:** mammary carcinoma, colon carcinoma, osteosarcoma, Jak-STAT1 signalling, microarray

## Abstract

The S100A4 protein has been associated with increased metastatic capacity of cancer cells, and recent studies have suggested a correlation between the expression level of S100A4 and the prognostic outcome for patients with various types of cancer. The knowledge about the mechanisms underlying the metastasis-promoting effects is still limited, and the aim of the present study was to elucidate signal transduction pathways involved in the regulation of S100A4. After treatment of human carcinoma cells with interferon-gamma (IFN-*γ*), we observed downregulation of S100A4 both at mRNA and protein levels. The effect was not dependent on IFN-*γ*-induced apoptosis or IFN-*γ*-mediated cell cycle arrest. Moreover, IFN-*γ*-mediated decrease in mRNA stability could not account for the observed decrease in S100A4 transcript level. Finally, microarray analysis suggests ISGF3G, ETV5, ZNF133 and CEBPG as possible candidate genes involved in IFN-*γ*-mediated repression of S100A4.

Several studies have associated the S100A4 protein with increased metastatic capacity of cancer cells (Davies *et al*, 1993; Mælandsmo *et al*, 1996; Levett *et al*, 2002), and more recently a correlation between the level of S100A4 protein and prognostic outcome has been reported in various types of cancer (lung gall bladder, oesophageal, gastric, breast and colon) (Kimura *et al*, 2000; Rudland *et al*, 2000; Yonemura *et al*, 2000; Ninomiya *et al*, 2001; Gongoll *et al*, 2002; Nakamura *et al*, 2002). Several laboratories have studied the involvement of S100A4 in invasion and metastasis, but little is still understood of the mechanism by which it exerts the metastasis-promoting effects. Previously, we reported an association between S100A4 expression, matrix metalloproteinase activity and the metastatic capacity of human osteosarcoma cells (Bjornland *et al*, 1999). Other studies have reported interactions between S100A4 and cytoskeletal associated proteins, suggesting a function in cell motility, thereby contributing to increased metastatic potential (Davies *et al*, 1993; Watanabe *et al*, 1993; Kriajevska *et al*, 1998,^2000^; Ambartsumian *et al*, 2001). In addition, Ambartsumian *et al* (2001) reported that when secreted from tumour cells, S100A4 could cause cancer progression through stimulation of angio-genesis. Finally, S100A4 has been found to interact with p53, S100A1 and, recently, liprin beta 1 (Wang *et al*, 2000; Chen *et al*, 2001; Kriajevska *et al*, 2002). Interaction with p53 would implicate a role for this S100 protein in fundamental cellular events like cell cycle regulation and apoptosis, while S100A4 interaction with liprin beta 1 points to a role in cytoskeletal dynamics and cell adhesion.

The aim of the present study was to identify mediators involved in the regulation of S100A4 expression, and to elucidate implicated signal transduction pathways. Several signal inducers were tested, but only interferon-gamma (IFN-*γ*) revealed any effects, resulting in reduction in S100A4 mRNA and protein levels in all tested cell lines. Upon cytokine binding, the IFN-*γ*-receptor subunits dimerise and thereby activate the intrinsic kinase leading to phosphorylation of the associated Jak-1 and Jak-2. These kinases subsequently phosphorylate STAT1, which when phosphorylated forms homodimers, translocates to the nucleus and activates transcription by binding to the interferon-gamma-activated sequence (GAS). This binding affects gene expression that ultimately accounts for the diverse cellular effects induced by IFN-*γ*, including the well-documented cell cycle arrest and/or apoptosis (reviewed by Boehm *et al*, 1997; Stark *et al*, 1998; Samuel, 2001).

The possibility that the observed regulation of S100A4 expression by IFN-*γ* was a secondary event caused by IFN-*γ*-induced cell cycle arrest or apoptosis was excluded, and indications for reduced transcription caused by upstream factors were obtained. In search for factors instrumental for the observed repression, microarray analysis revealed a number of candidate genes including ISGF3G, ETV5, ZNF133 and CEBPG.

## MATERIALS AND METHODS

### Materials

Interferon-gamma and thapsigargin were from Calbiochem (Nottingham, UK). PD98059 was from New England Biolabs (Beverly, MA, USA). Tumour necrosis factor-alpha (TNF-*α*), actinomycin D and transforming growth factor-beta (TGF-*β*) were all from Sigma Chemical Company (St Louis, MO, USA).

### Cell culture

All the examined cell lines (OHS (Fodstad *et al*, 1986), II-11b (anti-S100A4-ribozyme transfected OHS cells, Maelandsmo *et al*, 1996), WiDr (colon carcinoma, American Type Culture Collection, Manassas, VA, USA) and PM1 (breast carcinoma, established in our lab)) were cultivated in RPMI-1640 (Bio Whittaker, Verviers, Belgium) containing 10% fetal bovine serum (FBS; Biochrome KG, Berlin, Germany), 50 IE penicillin, 0.1 *μ*g ml^−1^ streptomycin (Bio Whittakker, Verviers, Belgium), 20 mM HEPES and 2.0 mM Glutamax (GIBCO BRL, Life Technologies, Paisley, UK). For all experiments, subconfluent cultures were trypsinated and seeded at 2 × 10^4^ cells cm^−2^ in 25 or 75 cm^2^ culture flasks, except for proliferation and apoptosis experiments where 5000 cells per well were seeded in 96-well cell culture plates. After overnight incubation, the culture medium was replaced with medium in the presence or absence of IFN-*γ* and harvested as indicated in the text.

### Northern blotting

Total RNA was isolated by a single-step RNA isolation method using Trizol® (GIBCO BRL, Life Technologies, Paisley, UK) according to the manufacturer's protocol. A measure of 5 *μ*g of total RNA was separated by electrophoresis through 1% agarose gels containing 6.6% formaldehyde in 0.02 M NaH_2_PO_4_ (pH 6.6) and transferred onto Hybond-N+ membranes (Amersham Pharmacia Biotech, Uppsala, Sweden). After baking for 2 h and subsequent UV crosslinking, filters were hybridised with DNA probes labelled with ^32^P by the random priming technique (Feinberg and Vogelstein, 1983). The hybridisations were carried out in 0.5 M Na_2_HPO_4_ (pH 7.2) and 1% SDS according to Church and Gilbert (1984). For multiple hybridisations, the bound probe was removed by incubating the filters twice for 5 min in 0.1 × SSC and 0.1% SDS at 95–100°C. To correct for an uneven amount of RNA loaded in each lane, filters were rehybridised with a kinase labelled oligonucleotide probe specific for human 18S rRNA. The mRNA levels were measured using a Storm PhosphoImage-scanner (Amersham Biosciences, Sunnyvale, CA, USA) and quantified using an ImageQuant software package (Amersham Biosciences, Sunnyvale, CA, USA). The cDNA probe for S100A4 was kindly provided by Dr CW Heizmann, University Hospital of Zürich, Switzerland.

### Western blotting

Protein lysates were prepared in 50 mM Tris-HCl (pH 7.5), containing 150 mM NaCl and 0.1% NP-40 with 2 *μ*g ml^−1^ pepstatin, aprotinin (Sigma Chemical company, St Louis, MO, USA) and leupeptin (Roche Diagnostics, Mannheim, Germany). Total protein lysate (40 *μ*g) from each sample was separated by 12% sodium dodecyl sulphate–polyacrylamide gel electrophoresis (SDS–PAGE), and transferred onto Immobilon-P membranes (Millipore, Bedford, MA, USA) according to the manufacturer's manual. As a loading and transfer control, the membranes were stained with 0.1% amidoblack. The membranes were subsequently incubated in 20 mM Tris-HCl (pH 7.5), containing 0.5 M NaCl and 0.25% Tween 20 (TBST) with 10% dry milk (blocking solution) before incubation with rabbit polyclonal anti-S100A4 (diluted 1 : 300, DAKO, Glostrup, Denmark), rabbit polyclonal anti-STAT1p91 (diluted 1 : 1000, Santa Cruz Biotechnology, Santa Cruz, CA, USA), and mouse monoclonal anti-alpha-tubulin (diluted 1 : 250, Amersham Life Science, Buckinghamshire, England) in TBST containing 5% dry milk. When immunostaining with rabbit polyclonal antiphospho-STAT1 (Tyr-701) (diluted 1 : 1000, Cell Signaling Technology, Beverly, MA, USA), TBST containing 0.05% Tween 20 was used. After washing, the immunoreactive proteins were visualised using horseradish peroxidase-conjugated secondary antibodies (diluted 1 : 5000 DAKO, Glostrup, Denmark), and the enhanced chemiluminescense system (Amersham Pharmacia Biotech, Buckinghamshire, England).

### Half-life of S100A4 protein

The half-life of the S100A4 protein was estimated by means of ^35^S-incorporation. The cell cultures were treated with methionine-free culture medium for 30 min before ^35^S-methionine (20 *μ*Ci ml^−1^, Amersham Pharmacia Biotech, Buckinghamshire, England) was added. After 1 h incubation, the medium was substituted with ordinary growth medium. Cells were harvested at different time points (0–48 h). Protein lysates were prepared and subjected to immunoprecipitation using 15 *μ*g rabbit polyclonal anti-S100A4 antibody. After collecting the immunoprecipitate with protein A-sepharose beads (Sigma-Aldrich, St Louis, MO, USA), the precipitates were resolved on a 12% SDS–PAGE gel. The dried gel was exposed for autoradiography overnight and the intensities of the S100A4 bands were subsequently analysed using a Personal Laser Densitometer SI (Amersham Biosciences, Sunnyvale, CA, USA).

### Cell proliferation

The proliferation in cell cultures was estimated by means of CellTiter 96® AQ_ueous_ One Solution Reagent (MTS) (Promega, Madison, WI, USA) according to the manufacturer's manual. The absorbance at 490 nm was recorded using a Wallac 1420 Victor^2^ Multilabel counter (Wallac Oy, Turku, Finland). All experiments were conducted at least in duplicate and repeated three times.

### Flow cytometry

Cell cycle distribution of control and IFN-*γ*-treated cell cultures were analysed by flow cytometry. The cultures were trypsinated, fixed in ice-cold methanol and stored at −20°C. Prior to analysis on a FACStar+ flow cytometer (Becton Dickinson, San Jose, CA, USA), cells were washed in phosphate-buffered saline (PBS) and resuspended in 2 *μ*g ml^−1^ Hoechst 33258 in PBS. Data analysis was carried out using the Modfit software (Verity Software House, Inc., Topsham, ME, USA). All experiments were conducted at least in duplicate and repeated three times.

### Apoptosis

The amount of cells undergoing programmed cell death was estimated using the Cell Death Elisa^Plus^ Kit (Roche Diagnostics, Mannheim, Germany), according to the manufacturer's manual. All assays were performed at least twice and in duplicate.

### Microarray analysis

Microarray analysis using in-house printed slides containing 11.5 k polymerase chain reaction (PCR)-amplified cDNAs was performed hybridising with cDNA probes prepared from control and IFN-*γ*-treated (10 U ml^−1^ for 48 h) OHS and II-11b cell cultures. Total RNA was isolated using the GenElute Mammalian Total RNA Kit (Sigma Chemical Co., St Louis, MO, USA). Each probe was made using Cy3-dUTP and Cy5-dUTP in cDNA labelling reactions starting with 30 *μ*g total RNA. Both untreated and IFN-*γ*-treated cultures were labelled with the two fluorophores and details for probe preparation and hybridisation conditions are included in the protocol located at http://www.med.uio.no/dnr/microarray. Totally, five hybridisations were performed (three OHS cDNA and two cDNA prepared from II-11b RNA), and the results are presented as average ratios from at least four hybridisations. Hybridised slides were scanned at a constant laser power of 100% for Cy3 and Cy5 and variable photomultiplier settings in a Scan Array 4000 instrument (GSI Lumonics, Inc., Billerica, MA, USA). Images obtained after scanning were imported to the GenePix Pro 3.0 software (Axon Instruments, Inc., Union City, CA, USA) for further analysis, including spot intensity measurements. Spots carrying technical-based artefacts were excluded manually.

## RESULTS

### Transcriptional regulation of S100A4

**Figure 1:**
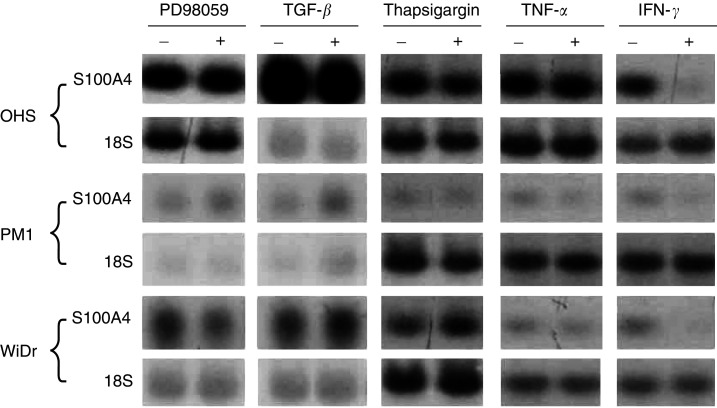
Representative Northern blots showing effects of different mitogens on the expression of S100A4. The OHS, PM1 and WiDr cell lines were incubated with 20 *μ*M PD98059 for 24 h, 0.2 nM TGF-*β* for 24 h, 0.5 ng ml^−1^ thapsigargin for 24 h, 10 ng ml^−1^ TNF-*α* for 48 h or 200 U ml^−1^ IFN-*γ* for 48 h.

**Figure 2:**
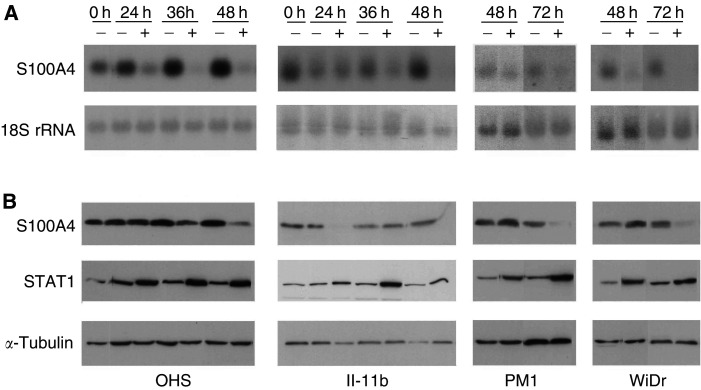
Optimisation of the IFN-*γ* response. (**A**) Northern blots showing transcriptional regulation of S100A4 in time–response experiments. OHS and II-11b cells were incubated with 10 U ml^−1^ IFN-*γ*, and WiDr and PM1 cells were incubated with 200 U ml^−1^ IFN-*γ*. Expression of 18S rRNA serves as loading control. (**B**) Western blots showing regulation of S100A4 and STAT1 by IFN-*γ* in time–response experiments. Incubation conditions as in (**A**) Immunostaining of *α*-tubulin serves as loading control.

In attempts to elucidate cellular pathways involved in the regulation of S100A4 gene expression, four cell lines with S100A4 protein levels varying from low (II-11b and PM1) to medium (WiDr) and high (OHS) were treated with different signal inducers. In initial screening experiments, effects were monitored at the transcriptional level in PM1, WiDr and OHS cells. Under the experimental conditions used, gene expression was not influenced by the MEK1-inhibitor PD98059, the calcium ionophore thapsigargin, TNF-*α* or TGF-*β* (Figure 1

). In contrast, IFN-*γ* induced a significant suppression of S100A4 transcription in all the cell lines (Figure 2A

), and 12–24 h later, a corresponding downregulation of S100A4 was observed at the protein level. As measured by densitometric scanning, the S100A4 mRNA level was reduced four-fold in the high expressing OHS cells, and three-fold in the II-11b, WiDr and PM1 cells (Figure 1 and Figure 2). The S100A4 response was observed at an earlier time point and at a lower dose in the osteosarcoma compared to the carcinoma cell lines (Figure 2A and B). Thus, maximal response was seen at 48 h with 10 U ml^−1^ in the OHS and II-11b cultures, whereas for PM1 and WiDr cells, 200 U ml^−1^ for 72 h was required to reach the same level of response. Notably, both WiDr and PM1 cells responded with induction of STAT1 after incubation with 50 U ml^−1^ IFN-*γ* for 48 h (results not shown), suggesting that the difference in S100A4 response could not be explained by increased sensitivity to IFN-*γ* in the osteosarcoma cells in general. The osteosarcoma cells did not show detectable downregulation of S100A4 at doses lower than 10 U ml^−1^ IFN-*γ*.

### Activation of the Jak/STAT1 signalling pathway

**Figure 3:**
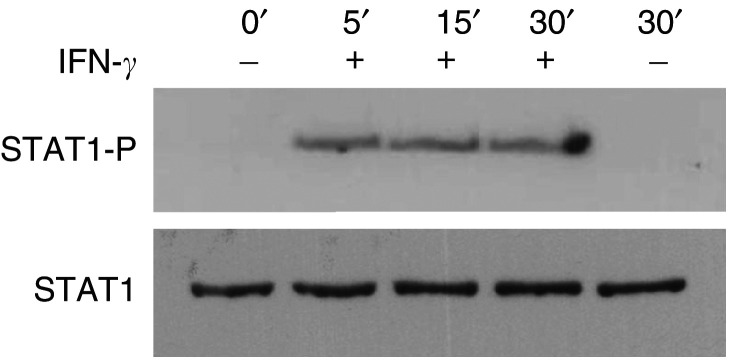
Confirmation of active Jak/STAT1 signalling pathway. Western blot stained with antiphospho-STAT1 (Tyr-701). OHS cells were treated with 1000 U ml^−1^ IFN-*γ* as indicated. Cells were harvested at indicated time points and analysed by Western blotting as described in Materials and Methods.

It is well documented that STAT1 gene expression is induced upon activation of the IFN-*γ*/Jak/STAT1 signalling pathway (Boehm *et al*, 1997) and, in agreement with this, an induction of STAT1 was observed in all the examined cell lines (Figure 2B). To further confirm activation of STAT1, lysates from IFN-*γ*-treated OHS cells were stained with antiphospho-STAT1 (Tyr-701) antibody (Figure 3

). The results showed that the treatment induced phosphorylation of STAT1 within a few minutes, thus confirming an active signalling pathway.

### Half-life of S100A4 protein

In order to explain the time gap between the observed downregulation of S100A4 mRNA and the corresponding decline at the protein level, we used a radioactive incorporation assay to determine the half-life of the protein. The estimated half-life of S100A4 in OHS was 24 h (results not shown), which might explain the observed lag period between mRNA and protein response (Figure 2A and B).

### Effects on cell proliferation and cell cycle distribution

As measured by the MTS assay, IFN-*γ* inhibited proliferation in OHS and WiDr cells by approximately 50%, while II-11b and PM1 cells were less influenced by the treatment, showing only 13 and 17% reduction in proliferation rate, respectively (Table 1

**Table 1:** Cell cycle distribution, proliferation and apoptosis in IFN-*γ* treated cell cultures

**Cell line**	**IFN-*γ* (U ml^−1^)**	**Hours of treatment**	**% cells in G0/G1 (s.d.)**	**% cells in S (s.d.)**	**% cells in G2/M (s.d.)**	**MTS assay (proliferation) % of control (s.d.)**	**Apoptosis fold induction (s.d.)**
OHS	—	0	46 (7)	16 (6)	38 (7)		
	—	48	55 (10)	11 (6)	34 (11)	100	1
	10	48	48 (3)	9 (4)	43 (7)	52 (15)	3.5 (2.4)

II-11b	—	0	38 (6)	4 (11)	51 (10)		
	—	48	48 (7)	15 (7)	37 (4)	100	1
	10	48	44 (6)	15 (2)	41 (5)	87 (13)	1.6 (0.6)

PM1	—	0	68 (4)	14 (5)	18 (1)		
	—	72	70 (8)	9 (5)	21 (4)	100	1
	200	72	72 (7)	8 (3)	20 (7)	83 (5)	1 (0.1)

WiDr	—	0	40 (12)	18 (6)	42 (11)		
	—	72	72 (7)	8 (4)	20 (7)	100	1
	200	72	66 (9)	5 (5)	29 (12)	53 (8)	5.7 (0.1)

). The cell cycle distribution of IFN-*γ*-treated cell cultures was analysed by flow cytometry. Both treated and untreated WiDr cells demonstrated an accumulation of cells in the G0/G1 phase. This may be explained by the long incubation period (72 h), while neither the treatment nor the long incubation time had any clear effect on cell cycle distribution in the OHS, II-11b or PM1 cell lines.

### IFN-*γ*-mediated induction of apoptosis

To study the possible effects of IFN-*γ* treatment on apoptosis, an immunoreagent-based apoptosis detection kit was utilised. A 3.5- and 5.7-fold increase in apoptosis was seen for the OHS and WiDr cell lines, respectively, as compared to the corresponding control cultures. For the low S100A4-expressing cell line II-11b, a 1.6-fold increase in apoptosis was found, whereas the fraction of apoptotic PM1 cells was unchanged by the treatment. These results are in agreement with the cell survival data. Thus, whereas the II-11b and PM1 were only slightly influenced by IFN-*γ*, OHS and WiDr cell survival was reduced to approximately 50% (Table 1). Notably, independent of the induction of apoptosis, S100A4 expression was downregulated by IFN-*γ* in all the cell lines.

### Analysis of S100A4 mRNA stability

**Figure 4:**
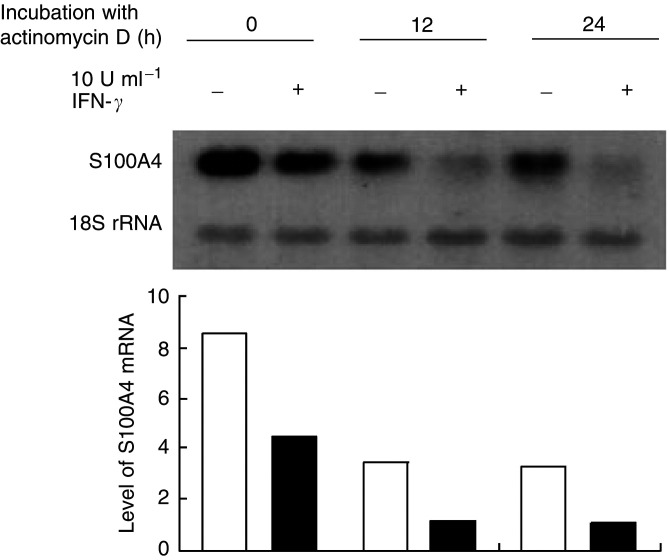
Effect of IFN-*γ* on S100A4 mRNA stability in OHS cells. Northern blot analysis demonstrating S100A4 mRNA stability in OHS control cultures and cultures treated with 10 U ml^−1^ IFN-*γ*. The cultures were incubated for 24 h before the addition of 5 *μ*M actinomycin D. The cells were then harvested at different time points as indicated. Isolation of total RNA and Northern blotting were performed as described in Materials and Methods. The chart is a graphical presentation of the band intensities as measured by means of densitometric scanning. The figure shows one of three independent experiments with similar results.

To study whether IFN-*γ*-mediated downregulation of S100A4 expression could be explained by decreased mRNA stability, OHS cells were incubated with IFN-*γ* for 24 h before adding actinomycin D, a general inhibitor of transcription. Cells were harvested at different time points (0–24 h) after the addition of actinomycin D, total RNA was extracted and the amount of S100A4 transcript was analysed by means of Northern blotting and densitometric scanning. As shown in Figure 4

, no significant differences in S100A4 mRNA decay between IFN-*γ*-treated and untreated cultures were demonstrated.

### IFN-*γ*-responsive genes as measured by cDNA microarray

To search for candidate genes involved in IFN-*γ*-mediated repression of S100A4 transcription, cDNA microarray analysis was performed, focusing on DNA-binding genes/transcription factors/repressors that were significantly up- or downregulated by the treatment. By defining three-fold increase/decrease in both cell lines as a cutoff level, four candidate genes (ISGF3G, ETV5, ZNF133, CEBPG) were identified (Table 2

**Table 2:** Summary of microarray results

**ID number**	**ID name**	**Common name**	**Function^a^**	**Ratio^b^**
724588	ISGF3G	Interferon-stimulated transcription factor 3 gamma, p48, IRF-9	Responsible for the initial stimulation of IFN-*α*- and *β*-responsive genes, it recognises and binds to the IFN-stimulated response element, or ISRE within the regulatory sequences of target genes. ISGF3 plays a primary role in the transmission of a signal from the cell surface to the nucleus	9
796542	ETV5	ERM, ETS-related protein	Binds to DNA sequences containing the consensus nucleotide core sequence ggaa	6
50794	ZNF133	Zinc-finger protein 133	May be involved in transcriptional regulation as a repressor	4
455121	CEBPG	CCAAT/enhancer binding protein gamma	Transcription factor that binds to the enhancer element PRE-i (positive regulatory element-i) of the IL-4 gene. Might change the DNA-binding specificity of other transcription factors and recruit them to unusual DNA sites	4

a Description of genes are modified from Rebhan *et al*. (1997). URL: http://bioinformatics.weizmann.ac.il/cards.

b OHS and II-11b cells treated with IFN-*γ* (10 U ml^−1^, 48 h) *vs* untreated control, average ratio from four hybridisations.

). All four cDNAs have been confirmed by sequencing, and the IFN-*γ*-mediated upregulation of all four genes as well as IFN-*γ*-mediated downregulation of S100A4 has been verified by Northern blotting. Whether any of these four factors are involved in IFN-*γ*-mediated repression of S100A4 expression is currently under investigation.

## DISCUSSION

### Transcriptional regulation of S100A4

Transcriptional regulation of S100A4 in response to treatment with different signal inducers was examined in cell lines expressing different levels of the protein. IFN-*γ*-mediated downregulation of S100A4 was observed in all the examined cell lines, but the sensitivity varied with respect to dose and time of incubation. When exposed to a low-dose IFN-*γ* (and for a shorter incubation period), STAT1 was induced in all four cell lines, indicating that the receptor is present, and that the extent of S100A4 transcriptional downregulation could not be attributed to a general difference in IFN-*γ* sensitivity.

Considering the fundamental biological effects induced by the MAP kinase cascade, and also by TGF-*β* and TNF-*α*, it was somewhat surprising that none of these signalling pathways were found to modulate the transcription of S100A4. Several studies have indicated that S100 proteins, due to their Ca^2+^-binding properties, are involved in regulating intracellular Ca^2+^ homeostasis or Ca^2+^-dependent cell signalling (Boni *et al*, 1997; Duarte *et al*, 1998). In addition, an intracellular relocation of S100A4 upon treatment with thapsigargin has been reported (Mueller *et al*, 1999). We did not, however, observe any changes in S100A4 expression caused by thapsigargin, indicating that no direct association between transcriptional regulation of S100A4 and Ca^2+^-dependent cell signalling existed in our cell systems. In agreement with our results, Takenaga (1999) showed that the expression of S100A4 in human colon adenocarcinoma cell lines was regulated by IFN-*γ*, but not influenced by IL-1, IL-6, various growth factors or IFN-*α* and -*β*. Taken together, the expression of S100A4 seems to be rather stable in cancer cells, and one might suggest that signalling pathways regulating S100A4 gene transcription in cultured cancer cells are well defined, without extensive crosstalk between the different pathways. Whether this is the case in cancer cells *in vivo* remains to be investigated.

### IFN-*γ* induced apoptosis but no cell cycle arrest

IFN-*γ* may inhibit cell proliferation and induce cell cycle arrest and/or apoptosis through diverse mechanisms. Since no differences in cell cycle distribution between treated and control cell cultures were revealed, we propose that downregulation of S100A4 expression could not be explained by IFN-*γ*-mediated cell cycle arrest.

Using an immunoreagent-based apoptosis detection kit, we found that the two cell lines, OHS (high expression of S100A4) and WiDr, were more susceptible to IFN-*γ*-induced apoptosis (up to five-fold increase) than the low S100A4 expressing II-11b and PM1 cell lines. Notably, the latter was completely resistant to IFN-*γ*-induced apoptosis. Recently, Grigorian *et al* (2001) reported a significant inverse correlation between expression of wild-type p53 and S100A4 in a panel of tumour cell lines, and suggested that S100A4 could cooperate with wild-type p53 in triggering apoptosis (Grigorian *et al*, 2001). PM1 is the only cell line in our study harbouring wild-type p53 (unpublished). According to our results, wild-type p53 combined with a low expression of S100A4 protected the cells against IFN-*γ*-mediated apoptosis. Interestingly, II-11b cells with a low expression of S100A4 were significantly less susceptible to IFN-*γ*-mediated apoptosis than the high expressing counterpart, OHS. Whether these differences in induction of apoptosis can be ascribed to the disparate expression level of S100A4 is currently under investigation. Importantly, expression of S100A4 was downregulated in all four cell lines, independent of the level of apoptosis. Based on this observation, we concluded that the observed repression of S100A4 was not caused by IFN-*γ*-mediated apoptosis.

### IFN-*γ* mediated no change in S100A4 mRNA stability

The observed IFN-*γ*-mediated downregulation of S100A4 might be caused by decreased mRNA stability, or alternatively by repression of transcriptional activity. In contrast to previous results (Takenaga, 1999), treatment of the OHS cells with actinomycin D revealed no significant difference in RNA stability upon the addition of IFN-*γ*. One possible explanation for this discrepancy is that different cell lines were used. We concluded that in OHS cells, IFN-*γ*-mediated downregulation of S100A4 was most likely caused by repression of S100A4 gene transcription and not a result of decreased mRNA stability.

### Candidate genes involved in repression of S100A4 transcription as revealed by microarray analysis

Our results indicated that the S100A4 promoter carries elements/regions that are susceptible to IFN-*γ*-mediated signalling. Notably, the time gap between phosphorylation of STAT1 protein (within a few minutes) and detectable reduction of S100A4 transcription (24 h) suggested that the latter response was not a direct effect of STAT1 binding to elements in the S100A4 promoter. In line with this, no IFN-GAS was found in the promoter comprising 2500 base pairs (bp) upstream of ATG. Several other proteins may interact with STAT1 and modulate its transcriptional activity, for example, the histone acetyltransferases CBP/p300 and the tumour suppressor BRCA1 (Zhang *et al*, 1996; Ouchi *et al*, 2000). It has also recently been published that IFN-*γ* may activate other signalling pathways, like the MAP kinases, independent of STAT1 activation (Gil *et al*, 2001; Ramana *et al*, 2001). This complex picture of proteins involved in the regulation of IFN-*γ*-induced gene expression led us to perform microarray analysis in an attempt to identify candidates participating in the observed repression of S100A4 gene expression. We identified four genes, of which ISGF3G (IRF-9/p48) was the most pronounced upregulated transcript (nine-fold), while a member of the CCAAT-enhancer-binding protein family, CEBP-gamma (CEBPG), was upregulated four-fold. Notably, it was recently shown that another member of the family (CEBPB) was transcriptionally induced by IFN-*γ*, and subsequently regulated the transcription of ISGF3G through the IFN-*γ*-activated transcriptional element (GATE) (Roy *et al*, 2002). Members of the CEBP family have been shown to interact with CBP/p300 and members of the Ets family of transcription factors (Mink *et al*, 1997; McNagny *et al*, 1998). ETV5, which is an Ets family member, was found to be upregulated more than six-fold in our IFN-*γ*-treated cells. ZNF133 has previously been associated with transcriptional repression in other cell systems (Vissing *et al*, 1995). We were, however, not able to find binding sites for ZNF133 or ISGF3G when examining 1000 bp upstream of the human S100A4 translation start site. In contrast, several potential binding sites for factors of the CEBP family, Ets family and p300 were detected (Schug and Overton, 1997).

In conclusion, we have found that IFN-*γ* downregulates S100A4 expression in osteosarcoma, breast and colon carcinoma cell lines. Our results indicate that this downregulation was mediated by inhibition of the S100A4 gene transcription, and not, as previously reported, caused by the IFN-*γ*-mediated decrease of S100A4 mRNA stability. S100A4 transcriptional activity was repressed independently of IFN-*γ*-mediated apoptosis and cell cycle effects. Therefore, we are currently investigating the S100A4 promoter in order to identify responsible transcriptional elements and mechanisms involved in IFN-*γ*-mediated inhibition of S100A4 gene transcription.
